# The Performance of Three Immune Assays to Assess the Serological Status of Cattle Experimentally Exposed to *Mycoplasma bovis*

**DOI:** 10.3390/vetsci5010027

**Published:** 2018-03-08

**Authors:** Meghan L. Schibrowski, Tamsin S. Barnes, Nadeeka K. Wawegama, Megan E. Vance, Philip F. Markham, Peter D. Mansell, Marc S. Marenda, Anna Kanci, José Perez-Casal, Glenn F. Browning, Justine S. Gibson, Timothy J. Mahony

**Affiliations:** 1 Queensland Alliance for Agriculture and Food Innovation, The University of Queensland, St Lucia, QLD 4072, Australia; meg_schib@hotmail.com (M.L.S.); t.barnes@uq.edu.au (T.S.B.); m.vance@uq.edu.au (M.E.V.); 2 School of Veterinary Science, The University of Queensland, Gatton, QLD 4343, Australia; gibson.j@uq.edu.au; 3 Asia-Pacific Centre for Animal Health, Faculty of Veterinary and Agricultural Sciences, The University of Melbourne, Parkville, VIC 3010, Australia; nadeekaw@unimelb.edu.au (N.K.W.); pmarkham@unimelb.edu.au (P.F.M); pmansell@unimelb.edu.au (P.D.M.); mmarenda@unimelb.edu.au (M.S.M.); akanci@unimelb.edu.au (A.K.); glenfb@unimelb.edu.au (G.F.B.); 4 Vaccine and Infectious Disease Organization-International Vaccine Centre (VIDO-InterVac), 120 Veterinary Road, Saskatoon, SK S7N 5E3, Canada; jose.perez-casal@usask.ca

**Keywords:** *Mycoplasma bovis*, serology, sensitivity, specificity, ELISA, Western blotting

## Abstract

*Mycoplasma bovis* is associated with several clinical syndromes of cattle. Currently, limited information is available on the sensitivity (*Se*) and specificity (*Sp*) of serological assays used for the detection of *M. bovis*-specific antibodies. Consequently, it is difficult to critically evaluate the outcomes of studies that use these assays. Therefore, the current study used bovine sera sourced from *M. bovis* exposure studies from three countries to estimate the *Se* and *Sp* of two commercial *M. bovis* enzyme-linked immunosorbent assays (ELISA), BIO K302 and BIO K260, and Western blotting. Western blotting had the highest *Se* estimate of 74% (95% confidence interval (CI): 16–98%), compared to the BIO K302: 47% (95% CI: 10–87%) and BIO K260: 28% (95% CI: 1–92%). However, for *Sp,* the BIO K302: 96% (95% CI: 87–99%) and the BIO K260: 100% (95% CI: 93–100%) out-performed Western blotting: 88% (95% CI: 56–98%). Western blotting was the best assay for detecting seroconversion, correctly identifying 61% (95% CI: 29–86%) of exposed animals compared to 35% for BIO K302 (95% CI: 21–54%) and 8% for BIO K260 (95% CI: 0–87%). While none of the methods assessed had high *Se* and *Sp*, the availability of these estimates will aid in the interpretation of studies that use these assays. The results of this study highlight the difficulties encountered when using serology to detect exposure to *M. bovis* in cattle.

## 1. Introduction

*Mycoplasma bovis* is an important pathogen of cattle which has been associated with a number of clinical conditions in calves and has increasingly been recognised as a contributor to respiratory disease in older cattle and bison, identifying it as an emerging bovine pathogen [1,2,3,4,5].

Serological methods such as enzyme linked immunosorbent assays (ELISA) and Western blotting enable the retrospective detection of pathogen specific antibodies in a host serum sample following exposure. A critical element in the development of these methods is the identification of an antigen(s) specific for the organism of interest to which the host generates specific and detectable immune responses. The antigen must be highly conserved among different isolates and strains if the assay is to have broad utility. The identification of suitable antigens for *M. bovis* serological studies has proven problematic due to antigenic variation among different isolates. Western blotting studies have identified the variable surface proteins (Vsps) as the immunodominant antigens recognised by the host humoral response during *M. bovis* infections [6,7,8]. In addition, it has been demonstrated that the antibody reactivity of three Vsps (VspA, VspB and VspC) was independent of the clinical manifestation, the geographical origin of the *M. bovis* isolate, the mode of infection, and the animal history [8]. The Vsps, or at least some members of the Vsp family, appear to be persistently expressed by *M. bovis* during infection and the immunodominant domains are highly conserved among strains and isolates [8]. However, the genes encoding the major immunological determinants have been shown to be subject to high frequency phase and antigenic variation [9]. This variation has the potential to adversely affect the reliability of diagnostic assays that utilise Vsp antigens [10]. Despite these concerns, it is possible that selected motifs of the Vsps could be useful for the development of *M. bovis* sero-diagnostic assays for epidemiological studies.

Approximately 80% of the VspA amino acid sequence is composed of two domains of repeated sequence, separated from each other by 22 amino acid blocks. The first block, localised at the N-terminal region, is composed of two distinct motifs, designated R_A_1 and R_A_2, while the second block, localised at the C-terminal region, contains three repeated motifs, R_A_3, R_A_4.1, and R_A_4.2 [8]. Homologues of the *vsp* gene were detected in 250 field isolates of *M. bovis* from France, Germany, Italy, Spain, and Switzerland, and all isolates were reported to contain multiple copies of the sequence encoding the R_A_1 motif [11]. These findings supported the conclusions of a previous study [8] and provided further evidence that the conserved domains within the Vsp family of proteins of isolates and/or strains could be used to improve *M. bovis* serological assays. The 106-amino acid R_A_1 motif within VspA contains PGENKT repeat elements and has been shown to be detected by antibodies induced following infection of cattle with *M. bovis* [6,8,12,13], suggesting the motif may be a useful candidate for inclusion in immunological assays. The immunodominant Vsp antigens of *M. bovis* can vary greatly both between and within strains and isolates and also exhibit high frequency phase variable expression [11]. Despite this, the R_A_1 motif of VspA has the potential to be a useful antigen for Western blot comparisons as it has been shown to be highly conserved and to elicit antibody responses detectable in the sera of cattle involved in outbreaks of *M. bovis* associated disease from geographically diverse regions [8,11].

Several studies have reported the use of non-commercial ELISAs to evaluate serological responses of cattle and related species in exposure trials [14,15,16,17,18,19,20]. Many of these assays used whole cell *M. bovis* antigen preparations, which can make comparisons of studies problematic. The potential variability in antigen expression by *M. bovis* isolates could adversely affect assay reproducibility and repeatability when antigens are prepared from whole cells [9].

Consequently, recent studies have attempted to identify antigens that could be over-expressed using recombinant DNA technology [21,22,23,24]. Of these antigens, only the assay using the MilA antigen [22] has been evaluated in a large number of field samples and had sensitivity (*Se*) and specificity (*Sp*) estimates reported [25]. Commercially available ELISAs with standardised production methods and standardised controls offer a potential solution to the variability between serological assays as all laboratories can access the same technology platform. However, these assays are still dependent on the test antigen(s) being universally expressed by the pathogen of interest. Typically, the details on the antigen(s) used in commercial ELISA are held as proprietary information. Users of these assays must accept the assumption that the population of interest was exposed to a *M. bovis* strain(s) that expresses the antigen(s) used in the ELISA.

Currently, there are few commercially available serological assays for the detection of antibodies against *M. bovis*. Several studies have reported serological data with respect to *M. bovis* using various non-commercial assays, making it difficult to compare studies [21,22,23,24]. For this reason, the current study has focused on commercially available assays, the BIO K302 and BIO K260, where standardisation of production and quality control processes, combined with general availability, could enable researchers to generate more comparable datasets. Two previous studies have estimated the *Se* and *Sp* of the BIO K302 assay, with one study also including the BIO K260 assay [25,26].

The aim of the current study was to estimate the *Se* and *Sp* of two commercially available *M. bovis* ELISA kits in parallel with Western blotting using panels of bovine reference sera from *M. bovis* experimental exposure studies from three countries. The capacity of the ELISA kits and Western blotting to detect seroconversion to *M. bovis* was also evaluated.

## 2. Materials and Methods 

### 2.1. Mycoplasma Culture and DNA Isolation

The *M. bovis* type strain PG45 in frozen modified Frey’s broth (MFB) was thawed on ice, streaked onto a modified Frey’s agar plate, and incubated at 37 °C in a 5% CO_2_ atmosphere for three to five days. A well isolated colony with typical *Mycoplasma* morphology was excised from the plate and used to inoculate 3 mL of MFB and the broth incubated for 48 h at 37 °C. The cells were recovered by centrifugation at 5500× *g* for 1 h at room temperature. The supernatant was aspirated, and the cell pellet washed twice with phosphate buffered saline (PBS). Genomic DNA was extracted from the cell pellet using the DNeasy Blood and Tissue Kit (QIAGEN, Hilden, Germany) according to the manufacturer’s instructions, quantified using a Nanodrop^®^ ND-1000 spectrophotometer (Thermo Fisher Scientific, Waltham, MA, USA) and stored at −20 °C until required.

### 2.2. Amplification and Cloning of the VspA R_A_1 Motif 

The *vspA* gene fragment encoding the R_A_1 motif [8] was amplified from *M. bovis* genomic DNA. The PCR reaction contained 20 mM Tris-HCl (pH 8.4), 50 mM KCl, 1.5 mM MgCl_2_, 0.2 mM dNTPs, 4 μg bovine serum albumin, 1 U Platinum^®^ *Taq* DNA polymerase (ThermoFisher Scientific), 8 pmol of each of the oligonucleotide primers *vspA*-F1 (5′-GTA GCA GCT AAA TGT GGT GAG ACC-3′) and *vspA*-R2 (5′-GTC ATC ATG CGG AAT TCT TGG-3′), and 25 ng of genomic DNA. The PCR cycling conditions utilised were: 94 °C for 3 min; 94 °C for 10 s, 54 °C for 15 s, and 72 °C for 30 s, 35 cycles; 72 °C for 2 min. The PCR amplicons were analysed by agarose gel electrophoresis through 1% (*w*/*v*) agarose gel at 7 V cm^−1^ for 1 h in 1 × TAE buffer (40 mM Tris-HCl pH 7.6, 20 mM acetic acid and 1 mM EDTA) and visualised by GelRed^TM^ staining and ultraviolet transillumination. The *vspA* amplicon was cloned into the pET SUMO™ plasmid vector using the Champion^TM^ pET-SUMO Protein Expression System (Thermo Fisher Scientific) as described by the manufacturer. The sequence and orientation of the *vspA*_R_A_1 amplicon in the pET-SUMO vector was confirmed using Big Dye Terminator v3.1 (Thermo Fisher Scientific, Waltham, MA, USA) sequencing according to the manufacturer’s instructions. The sequencing data were analysed using the DNASTAR™ suite of programs (DNASTAR, Madison, WI, USA). 

### 2.3. Expression of Recombinant Polypeptides

The SUMO vspA_R_A_1 fusion polypeptide (S-vspA_R_A_1) and control polypeptide SUMO-CAT were expressed using the plasmids pES_vspA_R_A_1-9 and pET SUMO/CAT (Thermo Fisher Scientific), respectively. The plasmids were used to transform chemically competent *Escherichia coli* One Shot^®^ BL21 (DE3) cells (Thermo Fisher Scientific) and express the polypeptide of interest following the manufacturer’s instructions. The bacterial cells were harvested by centrifugation at 5500× *g* for 10 min at 4 °C. The supernatant was carefully removed and discarded. The cell pellet was stored at −20 °C until required.

The frozen cell pellet was thawed on ice and suspended in 5 mL of cold *E. coli* Lysis Buffer^®^ (50 mM KH_2_PO_4_, pH 7.8, 400 mM NaCl, 100 mM KCl, 10% glycerol, 0.5% Triton X-100, 10 mM imidazole) with gentle agitation. Lysozyme was added to a final concentration of 250 μg mL^−1^ and benzonase nuclease to a final concentration of 5 U mL^−1^, and the mixture incubated at room temperature for 30 min with vigorous shaking. Following three freeze/thaw cycles, extracts were clarified at 5500× *g* for 10 min at 4 °C. The supernatant was carefully aspirated, and the polypeptide of interest purified using the Ni-NTA Spin-Kit (QIAGEN) according to the manufacturer’s instructions and stored at −20 °C until required. 

### 2.4. SDS-PAGE and Western Blotting

Protein samples of interest were prepared for SDS-PAGE analysis by mixing with an equal volume of sample reducing buffer (62.5 mM Tris-HCl pH 6.8, 10% glycerol, 2% (*w*/*v*) SDS, 6 mM dithiothreitol, 0.05% (*w*/*v*) bromophenol blue). The sample was gently vortexed, heated at 95 °C for 5 min, and then briefly centrifuged before being loaded onto the SDS-polyacrylamide gel. Electrophoresis was performed using a 0.75 mm thick 4% polyacrylamide stacking/10% polyacrylamide resolving gel in a Mini-PROTEAN™ Tetra Cell Electrophoresis Module (Bio-Rad Laboratories, Hercules, CA, USA) according to the manufacturer’s instructions. Following electrophoresis, the resolved polypeptides were transferred onto a nitrocellulose membrane for Western blot analyses using a mini-blot transfer apparatus (Bio-Rad Laboratories) according to the manufacturer’s instructions. The nitrocellulose membranes were blocked for 1 h at room temperature whilst shaking in blocking TBST buffer (20 mM Tris, 150 mM NaCl, 0.1% *v*/*v* Tween 20) containing 5% (*w*/*v*) skimmed milk powder (TBST-B). Primary antibodies were diluted in TBST-B and incubated overnight, with gentle agitation, at 4 °C, and the membranes washed three times in cold TBST for 5 min. Membranes were then incubated for 1 h at room temperature with shaking in the secondary antibody in TBST-B. The membranes were subsequently washed three times with cold TBST for 5 min. Antibody binding was detected using chemiluminescence (Pierce ECL Plus Western Blotting Substrate, Thermo Fisher Scientific) and radiographic film using a 10 min exposure time.

### 2.5. Antibodies and Bovine Sera Used in This Study

#### 2.5.1. Monoclonal Antibodies:

The reactivity of the expressed S-vspA_R_A_1 was assessed with monoclonal antibodies that were known to bind to various *M. bovis* Vsp proteins (Supplemental file Table S1). 

#### 2.5.2. Canadian Cattle Sera

Bovine sera from Canada were included in the study. These sera were sourced from six to eight month old beef calves (*n* = 10) from a farm in southern Saskatchewan as part of a *M. bovis* experimental exposure study [17]. The immunoreactivity of the Canadian serum samples has previously been reported as negative or positive (further classified as having low or high reactivity) for the *M. bovis* antibody using a whole cell ELISA (Table 1) [17]. Samples were collected immediately prior to *M. bovis* exposure (Day 0) and 68 days after exposure (Day 68). Nine Day 68 samples were available for the *Se* analyses and five Day 0 samples for the *Sp* analyses. Paired serum samples from four animals were used to evaluate seroconversion with the two commercial ELISAs and the Western blot assay.

#### 2.5.3. English Cattle Sera 

Bovine serum samples from the Animal and Plant Health Agency (APHA) in England were also included in this study. The sera were collected from calves (*n* = 5) sourced from a farm reported to be free from pathogens commonly associated with bovine respiratory disease, including mycoplasmas. The calves were a dairy-cross breed and were five to seven weeks of age. The calves were challenged with a known pathogenic strain of *M. bovis*. Most of the samples included in the current study were from two animals experimentally exposed to *M. bovis* and one animal that remained unexposed. Blood samples were collected from the calves prior to exposure (Day 0) and on Day 7, Day 14, Day 21, and Day 28 after exposure. Prior to the current study, the Day 0 and Day 28 samples had been tested using an in-house whole cell ELISA (Table 2) [27]. In addition, serum samples from two other calves were included in the current analyses: a ‘positive control’ and a ‘cut-off control’ used by the APHA for the interpretation of the in-house whole cell *M. bovis* ELISA. The Day 28 samples from the two exposed calves, positive control, and cut-off control samples (used to define the minimum reactivity of a positive sample) were included in the *Se* analyses of the two commercial ELISAs and the Western blot assay (*n* = 4). The Day 0 samples from the exposed and unexposed calves and the Day 28 sample from the unexposed calf were used for the *Sp* analyses (*n* = 4). The sequentially collected samples from the exposed and unexposed animals were used to evaluate the timing of seroconversion to *M. bovis* in the Western blot assay, while the Day 0 and Day 28 paired samples from the two exposed animals were used in the seroconversion analysis of the three tests compared in this study.

#### 2.5.4. Australian Cattle Sera

Paired serum samples from 35 Australian calves collected during an *M. bovis* exposure trial (five unexposed and 30 exposed) [22], were used in the current study. The sera were obtained from male Friesian-cross calves which were free of *M. bovis* prior to experimental infection, as determined by culture and quantitative real-time PCR of nasal and conjunctival swabs [28]. The serum samples were collected on the day of *M. bovis* exposure (Day 0) and 24 days after exposure (Day 24) [22]. After collection, the serum samples were analysed for *M. bovis*-specific antibodies using the MilA-based ELISA and designated as either positive or negative (Table 3) [22]. The Day 24 sera from the 30 exposed calves were used for the *Se* analyses of the two commercial ELISAs and the Western blot assay. All 35 Day 0 sera and the Day 24 sera from the five unexposed calves were used for the *Sp* analyses (*n* = 40). The paired sera from the 30 exposed calves were used in the seroconversion analyses of the three tests compared in this study.

#### 2.5.5. Control Sera from Commercial Serological ELISA Assays

A total of 18 bovine control sera from commercially available *M. bovis* serological ELISA kits (BIO K302 and BIO K260) and the pentavalent bovine respiratory pathogen serological ELISA kit (BIO K284) were used in this study (Bio-X Diagnostics, Jemelle, Belgium). Positive and negative control sera were available from three batches of the BIO K302 assay (Batch numbers: MYC14C04, MYC13K26, MYC13J16), and four batches of the BIO K260 assay (Batch numbers: SMYC14C04, SMYC13K26, SMYC13J16, SMYC09I28). Positive control sera were available for four batches of the BIO K284 assay (Batch numbers: IBRPM12F04, IBRPM12K08, IBRPM12F28, IBRPM09L01).

### 2.6. Commercial ELISA Assays

The bovine serum samples used in this study were tested using the BIO K302 and BIO K260 ELISAs according to the manufacturer’s instructions (Bio-X Diagnostics). The ELISA validation and interpretation were performed as recommended by the manufacturer. Briefly, the BIO K302 ELISA was used to classify samples as either negative or positive for antibodies specific for an *M. bovis* recombinant antigen, while the BIO K260 ELISA was used to categorise samples semi-quantitatively (0, +, ++, +++, ++++, or +++++) with respect to the concentration of antibodies specific for an *M. bovis* recombinant antigen. In the current study, the B260 ELISA results are reported as 0, 1, 2, 3, 4, and 5, which correspond to the 0, +, ++, +++, ++++, and +++++ categories, respectively. In some analyses, the results from the BIO K260 were dichotomised, with “0” deemed negative, and samples categorised as “1” or higher deemed positive. The identity of the antigen(s) used in these indirect ELISAs is held as propriety knowledge, but the manufacturer discloses that it is a recombinant antigen expressed in *E. coli*. 

### 2.7. Western Blot Analyses with the S-vspA_R_A_1 Polypeptide

The S-vspA_R_A_1 polypeptide was transferred onto a nitrocellulose membrane and blocked as described above. The membrane was cut into 3 mm strips. Each strip was probed, washed, and developed with the bovine serum sample of interest diluted 1:100 and the secondary anti-bovine IgG-HRP antibody diluted 1:20,000, as described above. Samples which had no observable reactivity on the Western blot were deemed negative. Samples with detectable reactivity on the Western blot were deemed positive.

### 2.8. Detection of Seroconversion to M. bovis

To assess the capacity of each assay to detect a change in serological status with respect to *M. bovis*, the following criteria were applied. Only animals with a negative test result for their Day 0 sample were included in the analyses for each assay. For the BIO K302 assay, if the post-exposure sample was deemed to be positive according to the manufacturer’s instructions, the animal was considered to have seroconverted. For the BIO K260 assay, if the post-exposure sample was categorised as “++” or higher according to the manufacturer’s instructions, the animal was considered to have seroconverted. If the post-exposure sample generated an observable reaction, it was deemed to be positive and the animal was considered to have seroconverted.

### 2.9. Onset of Detectable Antibodies to M. bovis Post-Exposure

The S-vsp_R_A_1 Western blot assay was used to evaluate the onset of a detectable serum antibody response using the sera from the English cattle. The samples collected from each animal on Day 0, Day 7, Day 14, Day 21, and Day 28 after exposure to *M. bovis* were used in this analysis. The samples were deemed negative or positive as described above.

### 2.10. Statistical Methods

All data were managed using the statistical package Stata/SE 12.1^®^ (Statacorp, College Station, TX, USA). Test *Se* and *Sp* and associated 95% confidence intervals (CI) were estimated, adjusting for clustering by source (Canada, England and Australia). As the control samples were sourced from the commercial ELISA kits, how they were generated is unknown, and it was hypothesised that inclusion of these samples was likely to bias the *Se* and *Sp* estimates. Therefore, the *Se* and *Sp* estimates were calculated following the removal of the Bio-X Diagnostics control samples from the datasets. The proportions of animals that were initially seronegative, and that were exposed and then seroconverted using the BIO K302 ELISA, BIO K260 ELISA, and the Western blot assay were estimated, again adjusting for clustering by source (Canada, England, and Australia).

## 3. Results

### 3.1. Expression of the vspA-R_A_1 Motif 

The PCR yielded an amplicon of approximately 300 bp in length and the amplicon was cloned into the pET-SUMO vector. Sequencing of the plasmid, pES_vspA_R_A_1-9, revealed that the PCR amplicon was 306 bp in length. The vspA-R_A_1 motif encoded by the reference sequence of the *M. bovis* PG45 type strain encodes 10 PGENKT repeat units, while the amplicon generated in this study encoded eight PGENKT repeat units [29]. Otherwise, the nucleotide sequence of the vspA-R_A_1 amplicon was identical to that of the *M. bovis* PG45 type strain reference sequence (data not shown). 

The nucleotide sequence analysis confirmed the in-frame cloning of the SUMO fusion prolypeptide and the vspA_R_A_1 reading frames (data not shown). The fused SUMO-vspA_R_A_1 open reading frame was 675 bp in length and was predicted to encode a 225 amino acid polypeptide with an estimated molecular mass of 25 kDa. Following expression and metal affinity chromatography, a highly pure polypeptide with a molecular mass of 35–37 kDa was evident on SDS-PAGE gels. This polypeptide was considered to be the SUMO polypeptide fused to the vspA_R_A_1 motif (S-vspA_R_A_1).

### 3.2. Immunoreactivity of the S-vspA_R_A_1 Recombinant Polypeptide

**Figure 1:**
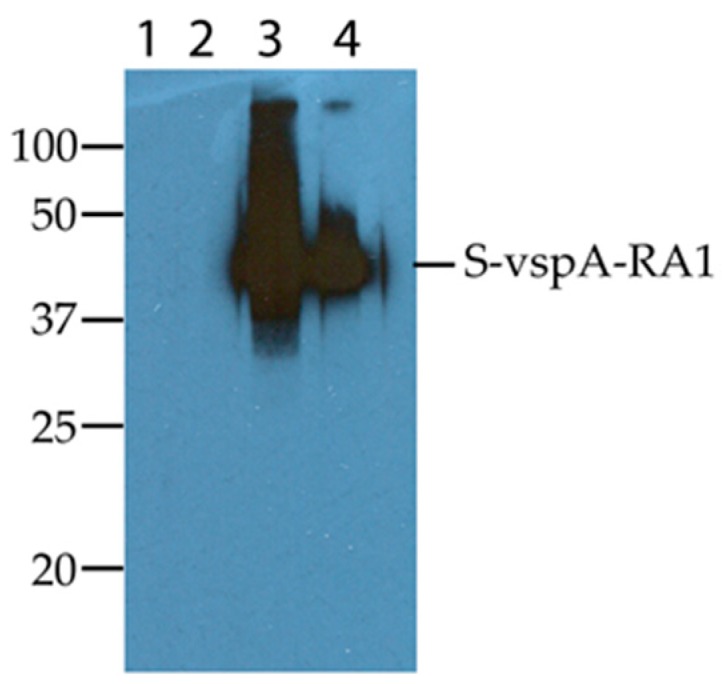
Reactivity of four monoclonal antibodies (MAb) with the S-vspA_R_A_1 polypeptide expressed in this study. The monoclonal antibodies bind to the variable surface lipoproteins of *Mycoplasma bovis*. Lane 1, MAb_1E5; Lane 2, MAb_9F1; Lane 3, MAb_4D7; Lane 4, MAb_6E5. The 100 kDa, 50 kDa, 37 kDa, 25 kDa, and 20 kDa molecular markers (kDa) are shown.

To compare the immunoreactivity of the S-vspA_R_A_1 recombinant polypeptide to the R_A_1 motif of native VspA, Western blot analyses were conducted using three VspA-reactive MAbs and two MAbs that bind to other *M. bovis* Vsps. Two of these antibodies, MAb_4D7 and MAb_6E5 [7], reacted with the S-vspA_R_A_1 recombinant polypeptide (Figure 1). In contrast, MAb_1E5 [7] did not show any reactivity with the recombinant protein (Figure 1). This result was expected as MAb_1E5 is reactive with an epitope in the C-terminal region of VspA, whereas the R_A_1 motif is located at the N-terminus of the protein [8,13]. The reactivity with MAb_4D7 and MAb_6E5 confirmed that the expressed polypeptide contained the VspA R_A_1 motif. The MAbs, MAb_2A8 [7] (data not shown) and MAb_9F1 [30], which do not bind VspA epitopes, did not react with the S-vspA_R_A_1 recombinant protein (Figure 1).

To assess whether the MAbs cross-reacted with the SUMO component of the fusion polypeptide, a second Western blot was performed with a SUMO-chloramphenicol acetyl transferase (SUMO-CAT) fusion protein produced in the same manner as the S-vspA_R_A_1 fusion protein. No immunoreactivity was detected between the SUMO-CAT fusion protein and these MAbs (data not shown).

### 3.3. Reactivity of Canadian Cattle Sera

- BIO K302 ELISA: All Day 0 samples (*n* = 5) from the Canadian cattle tested negative in the BIO K302 ELISA (Table 1), while seven of the nine Day 68 samples from animals exposed to *M. bovis* tested positive in this assay (Table 1).
- BIO K260 ELISA: All Day 0 samples (*n* = 5) from the Canadian cattle tested negative in the BIO K260 ELISA (Table 1), while six of the nine Day 68 samples from animals exposed to *M. bovis* tested positive in this assay (Table 1).
- Western blot: The Day 0 serum samples from Canadian cattle (*n* = 5) had varying reactivity with the S-vspA_R_A_1 recombinant polypeptide (Table 1). Three of the five Day 0 samples tested negative by Western blot, while the other two samples tested positive (Table 1). The Day 68 samples (*n* = 9) were reactive with the S-vspA_R_A_1 recombinant polypeptide and were deemed to be positive (Table 1).

### 3.4. Reactivity of English Cattle Sera

- BIO K302 ELISA: The Day 0 and Day 28 samples from the unexposed animal and the Day 0 samples from the two exposed animals tested negative in the BIO K302 ELISA (Table 2). Only one of the Day 28 samples from the two exposed animals tested positive (Table 2). The “cut-off control” and the “positive control” tested negative and positive, respectively (Table 2).
- BIO K260 ELISA: The Day 0 and Day 28 samples from the unexposed animal and the Day 0 samples from the two exposed animals tested negative in the BIO K260 ELISA (Table 2). Only one of the Day 28 samples from the two exposed animals tested positive (Table 2). The “cut-off control” and the “positive control” tested negative and positive, respectively (Table 2).
- Western blot: The immunoreactivities of the English cattle sera with the S-vspA_R_A_1 polypeptide from the two animals before and after exposure to *M. bovis* were consistent with the results of an in-house ELISA, with the Day 0 samples testing negative and the Day 28 samples testing positive (Table 2). The two control samples used in the in-house ELISA were both reactive in the Western blot assay and were deemed positive. Neither the Day 0 nor the Day 28 sample from the unexposed animal reacted with the S-vspA_R_A_1 polypeptide (Table 2).

### 3.5. Reactivity of Australian Cattle Sera

- BIO K302 ELISA: All samples (Day 0 and Day 24) from the group (*n* = 5) that were not exposed to *M. bovis* tested negative in the BIO K302 ELISA (Table 3). Two Day 0 samples from the *M. bovis* exposed group tested positive (APCAH32 and APCAH48) in this ELISA (Table 3). Eleven of the Day 24 samples from the exposed group (*n* = 30) tested positive in the BIO K302 ELISA (Table 3).
- BIO K260 ELISA: The Day 0 samples from the unexposed and exposed animals tested negative in the BIO K260 ELISA (Table 3). Four of the Day 24 samples from the exposed group (*n* = 30) tested positive in the BIO K302 ELISA (Table 3).
- Western blot: The S-vspA_R_A_1 Western blotting results were compared to the results of the MilA *M. bovis* ELISA (Table 3) [25]. None of the Day 0 or Day 24 serum samples from Australian calves (*n* = 5) that were not exposed to the *M. bovis* reacted with the S-vspA_R_A_1 (Table 3). Three of the Day 0 samples from the Australian calves that were subsequently exposed to *M. bovis* (*n* = 30) reacted with the S-vspA_R_A_1 polypeptide, while the remaining Day 0 samples did not react. The Day 24 samples from these animals also reacted in the Western blot. In total, 18 of the Day 24 samples from this group reacted with S-vspA_R_A_1 (Table 3).

To evaluate if any of the serum samples reactive with the S-vspA_R_A_1 were cross reacting with the SUMO fusion partner, sixteen of the paired sera (Day 0 and Day 24 from the same animal) were assessed for reactivity with the SUMO-CAT polypeptide using Western blotting (Supplemental file Figure S1; Table 3). Two and one of Day 0 and Day 24 sera from animals (*n* = 3) not exposed to *M. bovis* reacted with the SUMO-CAT polypeptide, respectively (Table 3). Four of the paired sera from animals (*n* = 13) exposed to *M. bovis* did not react with the SUMO-CAT polypeptide (Table 3). Five of the paired sera from animals exposed to *M. bovis* reacted with the SUMO-CAT polypeptide on both Day 0 and Day 24 (Table 3). Four of the paired sera were non-reactive on Day 0 and reactive on Day 24 (Table 3). 

### 3.6. Reactivity of Bio-X Diagnostics Control Sera

- BIO K302 ELISA: All the negative and positive controls provided in the BIO K302 and BIO K260 assays tested negative and positive, respectively, in the BIO K302 ELISA (Table 4). The *M. bovis* positive control samples from the BIO K284 assays all tested negative in this assay (Table 4).
- BIO K260 ELISA: All the negative and positive controls provided in the BIO K302 and BIO K260 assays tested negative and positive, respectively, in the BIO K260 ELISA (Table 4). The *M. bovis* positive control samples from the BIO K284 assays all tested negative in this assay (Table 4).
- Western blot: The reactivities of the ELISA control sera obtained from the three batches of the BIO K302 kit, the four batches of the BIO K260 kit, and the four batches of the pentavalent BIO K284 ELISA kit with the S-vspA_R_A_1 polypeptide are summarised in Table 4 (representative examples supplemental file Figure S1). The reactivity of all Bio-X Diagnostics sera with the SUMO-CAT polypeptide was also assessed. All positive control sera showed reactivity with S-vspA_R_A_1 and no reactivity was evident with any of the negative control sera tested (Table 4, representative examples supplemental file Figure S1). None of positive or negative sera from the BIO K302 or BIO K260 kits reacted with the SUMO-CAT polypeptide (Table 4). The positive control sera from the pentavalent BIO K284 reacted with the S-vspA_R_A_1 polypeptide and with the SUMO-CAT polypeptide (Table 4).

### 3.7. Immunoassay Se and Sp Estimates

The results for each of the immunoassays by animal exposure status and source are summarised in Table 5. These data were used to estimate the *Se* and *Sp* of each assay under evaluation in this study. The final datasets for each test contained 92 serum samples, from 43 exposed and 49 unexposed animals (Table 5). Using the final dataset, the BIO K302 test *Se* and *Sp*, adjusted for clustering by source, were estimated to be 47% (95% CI: 10–87%) and 96% (95% CI: 87–99%), respectively (Table 5). The BIO K260 test *Se* and *Sp* were estimated to be 28% (95% CI: 1–92%) and 100% (95% CI: 93–100%), respectively (Table 5).

Of the 43 sera from animals experimentally exposed to *M. bovis* that were tested in the Western blot assay, 31 showed reactivity with the S-vspA_R_A_1 fusion protein and were designated as seropositive for *M. bovis* (Table 5). The sera from 12 animals exposed to *M. bovis* had no detectable reactivity with the S-vspA_R_A_1 polypeptide. All were sourced from Australia (Table 5). In contrast, all the sera from animals exposed to *M. bovis* from Canada (*n* = 9) and England (*n* = 4) reacted in the Western blot (Table 5). The *Se* estimate for the Western blot assay, adjusted for clustering by source, was 72% (95% CI: 16–98%) (Table 5).

The Western blot assay was used to assess the sera of 49 animals that had not been exposed to *M. bovis*, and 44 had no reactivity with the S-vspA_R_A_1 polypeptide (Table 5). Five samples collected from animals prior to experimental exposure to *M. bovis* (Day 0) were reactive with the S-vspA_R_A_1 polypeptide. Two of these samples were from Canada (VIDO21 and VIDO30, Table 1), with the remaining three sourced from Australia (APCAH23, APCAH32, and APCAH48, Table 3). The *Sp* of the Western blot assay was estimated to be 90% (95% CI: 56–98%) (Table 5).

### 3.8. Detection of Seroconversion

In this study, 36 animals exposed to *M. bovis* had paired sera available for evaluating seroconversion (Table 1, Table 2 and Table 3). 

#### 3.8.1. Detection of a Seroconversion Using the BIO K302 ELISA 

Two Australian animals, APCAH32 and APCAH48, tested seropositive in the BIO K302 ELISA at Day 0 and were excluded from the analyses (Table 3). Of 34 remaining animals, 12 of the animals changed from seronegative to seropositive following experimental exposure to *M. bovis*, resulting in a seroconversion estimate of 35% (95% CI: 21–54%; Table 6). 

#### 3.8.2. Detection of Seroconversion Using the BIO K260 ELISA

The initial serum samples from all 36 animals experimentally exposed to *M. bovis* from which pre- and post-exposure serum samples were available were negative in the BIO K260 assay. Of these animals, three had a post-exposure BIO K260 assay result that was categorised as ‘++’ or higher and were deemed to have seroconverted, resulting a seroconversion estimate of 8% (95% CI: 0–87%; Table 6).

#### 3.8.3. Detection of Seroconversion Using Western Blotting

The capacity of the Western blot assay to detect seroconversion of animals exposed to *M. bovis* was also assessed using paired sera from the 36 experimentally exposed animals. As the Day 0 samples from two Canadian animals (Table 1) and three Australian animals (Table 3) reacted in the Western blot assay, these animals were excluded from the analysis. Of the remaining 31 animals, sera from 19 were positive after exposure to *M. bovis* in the Western blot assay and were deemed to have seroconverted, yielding a seroconversion estimate of 61% (95% CI: 29–86%; Table 6).

#### 3.8.4. Time to Serodetection

**Figure 2:**
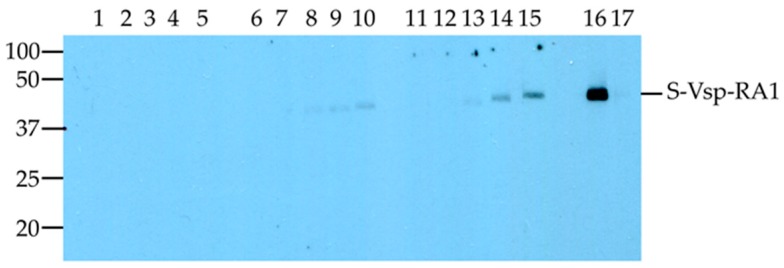
Western blot analysis of the reactivity of sera from English cattle with the S-vspA_R_A_1 recombinant polypeptide. The results for two exposed animals, AHVLA304 and AHVLA339, that were exposed to *Mycoplasma bovis* are shown. The serum samples were collected prior to *M. bovis* exposure, Day 0 (D0) and after exposure, Day 7 (D7), Day 14 (D14) Day 21 (D21), and Day 28 (D28). Lane 1, AHVLA370_D0; Lane 2, AHVLA370_D7; Lane 3, AHVLA370_D14; Lane 4, AHVLA370_D21; Lane 5, AHVLA370_D28; Lane 6, AHVLA304_D0; Lane 7, AHVLA304_D7; Lane 8, AHVLA304_D14; Lane 9, AHVLA304_D21; Lane 10, AHVLA304_D28; Lane 11, AHVLA339_D0; Lane 12, AHVLA339_D7; Lane 13, AHVLA339_D14; Lane 14, AHVLA339_D21; Lane 15, AHVLA339_D28; Lane 16, AHVLA ELISA positive control; Lane 17, AHVLA ELISA cut-off control. The 100 kDa, 50 kDa, 37 kDa, 25 kDa, and 20 kDa molecular markers (kDa) are shown.

The sera from the English exposure trial were used to estimate when *M. bovis*-specific antibodies could first be detected in exposed animals using the Western blot assay (Figure 2). For the two exposed animals, AHVLA304 and AHVLA339, Western blot reactivity was first detected on Day 14 for both animals (Figure 2). None of the serum samples collected from the animal (AHVLA370) that remained unexposed to *M. bovis* showed detectable reactivity in the Western blot assay (Figure 2). 

## 4. Discussion

The S-vspA_R_A_1 polypeptide expressed in this study migrated with an estimated molecular mass of 35 kDa in SDS-PAGE, even though it had a predicted molecular mass of 25 kDa. It has been reported that the Vsp antigens exhibit abnormal migration in SDS-PAGE gels [9]. The presence of a hexahistidine tag and repetitive amino acid motifs are likely to have resulted in the aberrant migration of the S-vspA_R_A_1 polypeptide using SDS-PAGE [31]. Importantly, the S-vspA_R_A_1 polypeptide was reactive with monoclonal antibodies specific for VspA, confirming that the presence of the SUMO fusion partner did not affect the antigenicity of the motif (Figure 1).

One potential source of non-specific reactivity in the Western blot assay described in this study was retention of the SUMO fusion partner in the S-vspA_R_A_1 polypeptide. To address this issue, the removal of the SUMO fusion partner was evaluated; however, this resulted in unworkable yields of the vspA_R_A_1 polypeptide (data not shown). Consequently, selected samples were analysed by Western blotting using the SUMO-CAT (Figure S1, Table 3 and Table 4). The observed reactivity of the samples was considered to be against the vspA_R_A_1 and CAT polypeptides and not the SUMO fusion partner for two reasons. Firstly, the sample reactivities (positive/negative) were not identical as would be expected if the antibodies were binding to the SUMO fusion partner, which was the common element in each assay. Secondly, analysis of the purified SUMO-CAT by Western blotting with an anti-hexa-histidine monoclonal antibody (Figure S1j, Lane 1), suggested that the polypeptide preparation contained a second smaller polypeptide considered to be unfused SUMO. While the proportion of the free SUMO was low, none of the reactive cattle serum or control samples had any detectable reactivity to this polypeptide (Figure S1). These results suggest that the observed reactivities of the cattle sera were not with the SUMO fusion partner and supported the use of S-vspA_R_A_1 as the antigen in the Western blot assay to evaluate *M. bovis* exposure status.

Of the sera collected from animals after exposure to *M. bovis*, all the Canadian and English sera were reactive with S-vspA_R_A_1 in Western blot assays (Table 1 and Table 2). In contrast, the post-exposure sera from the Australian cattle were less frequently reactive in the Western blot assay, with 60% (*n* = 18) testing positive (Table 3). There are several possible reasons why the post-infection sera sourced from Australia showed reduced reactivity with the S-vspA_R_A_1 compared to those sourced from England and Canada. 

The choice of *M. bovis* strains used in the three exposure studies may underlie the observed differences in these results. The panels of sera were generated in three geographically separate countries with different strains of *M. bovis*. There is currently limited information available about the immunogenicity of the *M. bovis* strain 3683 used in the Australian study [22], although a previous study has demonstrated it has the capacity to infect and cause disease in exposed calves [28]. Recently, the genomes of 82 Australian strains of *M. bovis* were analysed [32]. The genomic diversity between *M. bovis* strains was reported to be low and the strains examined had fewer *vsp* genes, including *vspA*, compared to the PG45 *M. bovis* reference strain [32]. However, as *M. bovis* strain 3683 was not included in the study, the frequency of the *vsp* genes in this strain remains to be determined. The Western blot results of the current study strongly suggest the 3683 strain encodes and expresses VspA or a related polypeptide which includes the R_A_1 motif. If the *M. bovis* strain 3683 strain expresses the R_A_1 motif at low levels or lacks VspG, VspH, and VspO, all of which can contain multiple repeats of the PGENKT amino acid domain [9], or expresses these antigens at reduced levels, then antibodies that react with the R_A_1 motif could be less abundant in the sera of exposed cattle or take longer to develop, thus explaining why fewer of the Australian animals were seropositive after exposure (Table 3). 

The complexity of *M. bovis* serology is exemplified by the Australian animals APCAH7, APCAH8, and APCAH18, as the Day 0 samples for these animals tested negative in all three assays (Table 3). In the MilA and the BIO K260 ELISAs, the Day 24 samples were also negative (Table 3). However, the Day 24 samples for these animals were all positive by Western blotting and APCAH8 was also positive using the BIO K302 ELISA (Table 3). These results suggest that the likelihood of an animal testing positive after *M. bovis* exposure may be influenced by the timing of the immune response to some antigens. Of the three groups of animals analysed in this study, the 24 day interval between exposure and the final sample collection for the Australian group was the shortest, compared to 28 days and 68 days for the English and Canadian samples, respectively. 

However, in the current study, specific antibodies to the R_A_1 motif were detected as early as Day 14 post exposure using the Western blot assay probed with the samples from the English cattle (Figure 2). Importantly, in both animals exposed to *M. bovis*, the level of antibody increased over time, suggesting that the reaction detected by the Western blot was specific to the R_A_1 motif. The detection of *M. bovis*-specific IgG at Day 14 is consistent with a previous study which detected IgG 10 to 14 days after experimental exposure [33]. Other reports have suggested that specific antibody responses can be detected 14 to 28 days after an invasive infection [21,34]. Animal factors, such as genetic background and age, may also have influenced when the calves used in these exposure studies developed detectable antibodies to the antigens used in each assay.

In the current study, the *Se* estimates for the two commercial serological assays were poor (Table 5). A previous report also estimated the *Se* and *Sp* of the BIO K302 (32%, 95% CI: 22–54% and 95%, 95% CI: 83–99%) and BIO K260 (13%, 95% CI: 51–30% and 100% CI: 77–96%) assays [25]. These estimates generally agree with the crude *Se* and *Sp* estimates for the Australian samples in the current study, a finding that was not unexpected as there is some overlap between the samples used (Table 5). The combined *Se* estimates for the three populations in the current study were very imprecise, when adjusted for clustering, as evidenced by the wide 95% CI (Table 5), and the different performances in each population (Table 5). However, the small number of samples from Canada and England prevented further investigation of this observation. Evaluation of the assays with a larger number of samples from these and other geographic regions will be required to fully understand this aspect of the performance of these tests.

Another study estimated the median *Se* and *Sp* of the BIO K302 ELISA to be 60% (38–96 95% Posterior Credibility Interval) and 97% (94–100 95% PCI), respectively, using the manufacturer’s recommended cut-off point [26]. These results suggest the commercial ELISA kits have good *Sp* and are unlikely to yield false positives. The authors of the study suggested increasing the manufacturer’s recommended cut-off point for the BIO K302 ELISA to reduce the likelihood of false positives [26]. The influence of this suggestion on the *Se* and *Sp* estimates in the current study was not investigated because of the smaller sample size.

Test *Se* was low for all three methods assessed in this study. Several post-exposure sera from Australia yielded negative results across all three methods, with 63% (*n* = 19), 87% (*n* = 26), and 40% (*n* = 12) of samples yielding ‘false negative’ results in the BIO K302 ELISA, the BIO K260 ELISA, and the Western blot assay, respectively (Table 3). In addition, two of the four English post-exposure sera and the cut-off control yielded ‘false negative’ results in the BIO K302 and BIO K260 ELISAs (Table 2), while ‘false negative’ results were also seen with the Canadian post-exposure samples using the BIO K302 (*n* = 2) and BIO K260 (*n* = 3) ELISAs (Table 1). The Canadian post-exposure sera that yielded negative ELISA results were classified as low sero-converters to *M. bovis* using an in-house ELISA [17].

The usefulness of the BIO K302, BIO K260, and Western blot assays for the detection of seroconversion using paired sera samples from animals was also assessed (Table 6). In the current study, sero-conversion was detected in 35% and 8% of paired animal sera using the BIO K302 and BIO K260 ELISAs, respectively (Table 6). These low levels of seroconversion were unexpected, as the BIO K260 ELISA is specifically marketed to detect seroconversion. Similarly, although the BIO K302 is not marketed to detect seroconversion, it would be expected that a sample collected from an animal following experimental exposure to *M. bovis* would yield a positive result in this assay. The low level of seroconversion detected in the current study was largely a result of a high proportion of Australian post-exposure samples failing to exceed the cut-offs assigned by the manufacturer of the BIO K302 and BIO K260 ELISAs (Table 3). However, only 50% of the samples from the Canadian (*n* = 4) and English (*n* = 2) animals from which paired sera were available were considered to have seroconverted post-exposure, so the source of samples and strain used for infection would not appear to be the sole explanation for these results. The Western blot assay performed considerably better for the detection of seroconversion, with 61% of exposed animals deemed to have seroconverted. As with the other assays, this result was strongly influenced by the Australian samples. The Western blot would not be suitable for testing large numbers of samples, but could be useful for testing a low number of samples such as in experimental trials or testing high value animals such as breeding bulls to establish or maintain *M. bovis*-free herds.

The results of this study highlight the difficulty of developing a serological assay that can be generally applied in all geographic regions for *M. bovis* serology. It has been proposed that a novel ELISA assay using the recently identified *M. bovis* antigen, MilA, could address this issue [22]. In the context of a recent study [32], which reported a reduction in the number of *vsp* genes in some Australian isolates examined, the next stage in the development of the MilA ELISA will be testing sera from cattle exposed to *M. bovis* strains sourced from different geographical regions. Similarly, the pyruvate dehydrogenase E1 component beta subunit has been identified as a promising *M. bovis* ELISA antigen, that when used in an ELISA, detected a higher number of positive serum samples compared to a commercial ELISA (*Mycoplasma bovis* Antibody Test Kit, BioVet, Saint-Hyacinthe, Canada) [24]. The panel of bovine sera used to compare the assays was from field samples and while some animals were reported to be clinically affected by *M. bovis*, the lack of knowledge of the true exposure status to the pathogen makes it difficult to assess the true performance of this assay [24]. The development of a competitive ELISA for *M. bovis* serology, based on the P48 lipoprotein, has also been reported [23]. The P48 ELISA detected higher numbers of serologically positive cattle compared to two commercially available ELISAs, the BioVet assay and an undisclosed Bio-X Diagnostics assay. As the relative performance of the assays was evaluated using the number of positives detected in field samples rather than *Se* and *Sp* estimations, true assay performance is difficult to assess. In light of these studies, the application of any serological test for *M. bovis* is likely to require some knowledge of antigen genomics to support the use of a specific antigen in any assay. Information on the *M. bovis* antigenic repertoire for the region of interest could also be used to modify assay components, including multiple recombinant antigens, to match the circulating *M. bovis* strains and improve assay performance.

As the manufacturer of the commercial ELISAs evaluated in this study holds the identity of the recombinant antigen as proprietary knowledge, it is not possible to specifically evaluate the utility of this antigen in a geographically diverse range of *M. bovis* strains. The reactivity of the Canadian and English samples suggests some utility in their application, but this is less evident when the Australian samples are also included. If the manufacturers of *M. bovis* serological assays wish to hold the assay components as proprietary knowledge, then they should also consider providing some additional support to potential users of these assays. This could include providing reagents or services to examine the levels of expression of the target antigen in *M. bovis* isolates from the region of interest to ensure some degree of confidence in the applicability of the assay and the validity of results. In the absence of this type of supporting information, potential users should carefully consider how the assay is used.

A recognised limitation of the current study is that samples collected from experimentally exposed animals are unsuitable for estimating *Se* and *Sp* of diagnostic assays due to a lack of independence of observations and variation in the immunological responses of experimentally infected animals compared to naturally infected animals [35]. The higher doses of *M. bovis* that calves may be exposed to in experimental infection may inflate the *Se* estimate over what might be seen in the field, and the absence of infection with related commensal mycoplasma species in experimental animals may inflate the *Sp* estimate. While the current study technically violates these ideal conditions, it has utilised samples from experimentally exposed animals from independent exposure experiments with several differing parameters (*M. bovis* strain, cattle age, and interval of sample collection), albeit in low numbers in two experiments. Consequently, the panel of sera may provide a more representative assessment of assay performance compared to sera sourced from a single exposure study. Despite this, the next stage in the assessment of these serological assays would be to estimate the *Se* and *Sp* using a panel of serum samples from validated field cases of *M. bovis* infection, when such a panel becomes available.

## 5. Conclusions

None of the serological assays evaluated in this study had 100% *Se* and 100% *Sp* for the detection of the *M. bovis* antibody in bovine serum. Although the Western blot assay had the highest combined estimates for *Se* and *Sp*, it is not useful as a screening test for large numbers of samples. The BIO K260 ELISA had the highest *Sp* estimates of the three methods assessed. While the point *Sp* estimate was 100%, the wide 95% confidence intervals suggest that the true *Sp* could be lower. The BIO K302 ELISA had the highest *Se* of the two ELISA kits, but was still quite low. The overall usefulness of the two commercial ELISAs evaluated in this manuscript will be dependent on the specific aims of the study. If the aim is to identify *M. bovis*-free herds, then a test that has a high *Se* and produces few false negatives is required. Based on the results of this study, neither the BIO K302 nor the BIO K260 ELISA would be useful for this purpose. If the aim is to identify the presence of *M. bovis* within a herd, then high *Sp* is required and either the BIO K302 or BIO K260 would be suitable, although the results would need to be interpreted in the knowledge that neither test would detect high numbers of positive animals in the study population, so the true prevalence of *M. bovis* is likely be underestimated. Knowledge of these limitations will enable the application of these assays, particularly within discrete populations, to better understand the biology of this important bovine pathogen.

## Figures and Tables

**Table 1:** The reactivity of Canadian cattle sera in the BIO K302 (B302), BIO K260 (B260), and Western blot (WB) *Mycoplasma bovis* serological assays. Serum was collected on Day 0 prior to and on Day 68 after experimental exposure to *M. bovis*. The results are shown as negative (Neg) or positive (Pos).

	Serological Assay							
	B302		B260 ^1^		WB		wcE ^2^	
Animal	Day 0	Day 68	Day 0	Day 68	Day 0	Day 68	Day 0	Day 68
VIDO18		Pos		Pos (5)		Pos		Pos (H)
VIDO19	Neg	Neg	Neg (0)	Neg (0)	Neg	Pos	Neg	Pos (L)
VIDO20	Neg	Pos	Neg (0)	Pos (2)	Pos	Pos	Neg	Pos (H)
VIDO21	Neg	Neg	Neg (0)	Neg (0)	Neg	Pos	Neg	Pos (L)
VIDO25		Pos		Pos (5)		Pos		Pos (H)
VIDO26		Pos		Neg (0)		Pos		Pos (L)
VIDO29		Pos		Pos (4)		Pos		Pos (H)
VIDO30	Neg	Pos	Neg (0)	Pos (3)	Pos	Pos	Neg	Pos (H)
VIDO34		Pos		Pos (3)		Pos		Pos (H)
VIDO38	Neg		Neg (0)		Neg		Neg	

^1^ The categorical values assigned to each sample are shown in parentheses. ^2^ The reactivity of serum samples tested using a *M. bovis* whole cell ELISA (wcE) as previously reported [17]. Positive samples were designated as either high (H) or low (L) reactors.

**Table 2:** The reactivity of English cattle sera in the BIO K302 (B302), BIO K260 (B260), and Western blot (WB) *Mycoplasma bovis* serological assays. Serum was collected from animals, prior to, Day 0, and after, Day 28, experimental exposure to *M. bovis*. The results are shown as negative (Neg) or positive (Pos).

	Serological Assay							
	B302		B260 ^1^		WB		wcE ^2^	
Animal	Day 0	Day 28	Day 0	Day 28	Day 0	Day 28	Day 0	Day 28
APHA370 ^3^	Neg	Neg	Neg (0)	Neg (0)	Neg	Neg	Neg	Neg
APHA304 ^4^	Neg	Neg	Neg (0)	Neg (0)	Neg	Pos	Neg	Pos
APHA339 ^4^	Neg	Pos	Neg (0)	Pos (1)	Neg	Pos	Neg	Pos
C_Cont ^2^		Neg		Neg (0)		Pos		Pos
P_Cont ^2^		Pos		Pos (4)		Pos		Pos

^1^ The categorical values assigned to each sample are shown in parentheses. ^2^ The reactivity of serum samples tested using a *M. bovis* whole cell ELISA (wcE) as previously reported whole cell ELISA [27]. Cut-off Control (C_Cont) and Positive Control (P_Cont) samples used in the wcE. ^3^ Animal not exposed to *M. bovis.* ^4^ Animal exposed to *M. bovis.*

**Table 3:** The reactivity of Australian cattle sera in the BIO K302 (B302), BIO K260 (B260), and Western blot (WB) serological assays. Samples were deemed negative (N) or positive (P) according to the criteria for each assay. Cattle were bled prior to exposure to *Mycoplasma bovis* on Day 0 (D0) and on Day 24 (D24) after exposure. Cattle were either remained unexposed or experimentally exposed to *M. bovis*.

	Serological Assay									
	B302		B260 ^1^		WB1 ^2^		WB2		MilA ^3^	
Animal	D0	D24	D0	D24	D0	D24	D0	D24	D0	D24
Unexposed										
APCAH13	N	N	N (0)	N (0)	N	N	P	P	N	N
APCAH24	N	N	N (0)	N (0)	N	N	P	N	N	N
APCAH30	N	N	N (0)	N (0)	N	N	N	N	N	N
APCAH43	N	N	N (0)	N (0)	N	N			N	N
APCAH71	N	N	N (0)	N (0)	N	N			N	N
Exposed										
APCAH3	N	N	N (0)	N (0)	N	N	N	N	N	P
APCAH5	N	P	N (0)	N (0)	N	P	N	P	N	P
APCAH7	N	N	N (0)	N (0)	N	P	N	N	N	N
APCAH8	N	P	N (0)	N (0)	N	P	N	N	N	N
APCAH9	N	P	N (0)	P (1)	N	P	P	P	N	P
APCAH10	N	N	N (0)	N (0)	N	P	P	P	N	P
APCAH18	N	N	N (0)	N (0)	N	P	P	P	N	N
APCAH20	N	P	N (0)	N (0)	N	P	N	P	N	P
APCAH21	N	N	N (0)	N (0)	N	P	N	P	N	P
APCAH22	N	P	N (0)	P (2)	N	P	N	N	N	P
APCAH23	N	N	N (0)	N (0)	P	P	P	P	N	P
APCAH26	N	P	N (0)	N (0)	N	P	P	P	N	P
APCAH31	N	N	N (0)	N (0)	N	N	N	P	N	P
APCAH32	P	P	N (0)	P (1)	P	P			N	P
APCAH34	N	N	N (0)	N (0)	N	N			N	P
APCAH35	N	P	N (0)	P (1)	N	P			N	P
APCAH36	N	P	N (0)	N (0)	N	N			N	P
APCAH45	N	P	N (0)	N (0)	N	N			N	P
APCAH46	N	N	N (0)	N (0)	N	N			N	P
APCAH48	P	P	N (0)	N (0)	P	P			N	P
APCAH49	N	N	N (0)	N (0)	N	N			N	P
APCAH51	N	N	N (0)	N (0)	N	N			N	P
APCAH52	N	N	N (0)	N (0)	N	N			N	P
APCAH53	N	N	N (0)	N (0)	N	N			N	P
APCAH54	N	N	N (0)	N (0)	N	P			N	P
APCAH57	N	N	N (0)	N (0)	N	P			N	P
APCAH60	N	N	N (0)	N (0)	N	N			N	P
APCAH62	N	N	N (0)	N (0)	N	P			N	P
APCAH63	N	N	N (0)	N (0)	N	N			N	P
APCAH64	N	N	N (0)	N (0)	N	P			N	P

^1^ The categorical values assigned to each sample are shown in parentheses. ^2^ Result in Western blot assay (WB1) with S-vspA_R_A_1 polypeptide; Western blot assay (WB2) with SUMO-CAT polypeptide (selected samples only). ^3^ Results from the MilA ELISA as previously reported [25].

**Table 4:** Reactivity of the negative control (NegC) and positive control (PosC) control samples from various Bio-X Diagnostics kits in BIO K302 (B302), BIO K260 (B260), and Western blot (WB) serological assays. Samples were deemed positive (P) or negative (N) according to the criteria for each assay. The batch numbers (Batch) for the kits from which the sera were sourced are also shown.

		Serological Assay							
		B302		B260 ^1^		WB1 ^2^		WB2 ^2^	
Assay	Batch	NegC	PosC	NegC	PosC	NegC	PosC	NegC	PosC
B302	MYC14C04	N	P		P (3)	N	P	N	N
	MYC13K26	N	P	N (0)	P (3)	N	P	N	N
	MYC13J16	N	P	N (0)	P (3)	N	P	N	N
B260	SMYC14C04	N	P	N (0)	P (3)	N	P	N	N
	SMYC13K26	N	P	N (0)	P (4)	N	P	N	N
	SMYC13J16	N	P	N (0)	P (3)	N	P	N	N
	SMYC09I28	N	P	N (0)	P (3)	N	P	N	N
B284	IBRPM12F04		N		N (0)		P		P
	IBRPM12K08		N		N (0)		P		P
	IBRPM12F28		N		N (0)		P		P
	IBRPM09L01		N		N (0)		P		P

^1^ The categorical values assigned to each sample are shown in parentheses. ^2^ Result in Western blot assay (WB1) with S-vspA_R_A_1 polypeptide; Western blot assay (WB2) with SUMO-CAT polypeptide.

**Table 5:** Summary of the results of *Mycoplasma bovis* serological assays used to estimate test sensitivity (*Se*) and specificity (*Sp*). The bovine sera testing using the BIO K302, dichotomised BIO K260, and Western blot are shown by source. The number (*n*) of samples in the *Se* and *Sp* analyses are shown as positive and negative, respectively, by source with the percentages (%) in parentheses. The *Se* and *Sp* estimates along with the 95% confidence intervals (CI) for all assays in parentheses.

		BIO K302	BIO K260	Western Blot
Source	*n*	No. Positive (%)	No. Positive (%)	No. Positive (%)
Sensitivity				
Canada	9	7 (78)	6 (67)	9 (100)
England	4	2 (50)	2 (50)	4 (100)
Australia	30	11 (37)	4 (13)	18 (60)
Total	43	20 (47%; 10–87%)	12 (28%; 1–92%)	31 (72%; 16–98%)
Specificity		N Negative (%)	N Negative (%)	N Negative (%)
Canada	5	5 (100)	5 (100)	3 (60.0)
England	4	4 (100)	4 (100)	4 (100)
Australia	40	38 (95.0)	40 (100)	37 (92.5)
Total	49	47 (96%; 87–99%)	49 (100%; 93–100%)	44 (90%; 56–98%)

**Table 6:** Detection of serconversion to *Mycoplasma bovis* using the BIO K302 ELISA, the BIO K260 ELISA, and the Western blot assay in experimentally exposed animals. The number of samples from seronegative (SNeg) animals exposed to *M. bovis* are shown. The number of these animals that seroconverted (SConv) after exposure is shown. The percentage that seroconverted and the 95% confidence intervals (CI) are shown in parentheses for each assay.

	BIO K302		BIO K260		Western Blot	
Source	SNeg	SConv	SNeg	SConv	SNeg	SConv
Canada	4	2 (50)	4	2 (50)	2	2 (100)
England	2	1 (50)	2	0 (0)	2	2 (100)
Australia	28	9 (32)	30	1 (13)	27	15 (56)
Total	34	12	36	3	31	19
		35% (CI: 21–54%)		8% (CI: 0–87%)		61% (CI: 29–86%)

## Supplementary Materials

The following are available online at http://www.mdpi.com/2306-7381/5/1/27/s1, Figure S1: Western blot analyses of selected Australian paired sera (*n* = 16) and ELISA control samples using the S-vspA-RA1 and SUMO-CAT polypeptides. Animal numbers are shown with postscripts indicated the day of serum collection, Day 0 (D0) and Day 24 (D24). The 100 kDa, 50 kDa, 37 kDa, 25 kDa, and 20 kDa molecular markers (kDa) are shown. Table S1: Monoclonal antibodies used in this study and the *Mycoplasma bovis* variable surface proteins (Vsp) recognised. The reported molecular sizes of each Vsp are shown in parentheses.
