# Supplementary material for: The Performance of Three Immune Assays to Assess the Serological Status of Cattle Experimentally Exposed to Mycoplasma bovis
Source: Vet Sci. 2018 Mar 8;5(1):27. doi: 10.3390/vetsci5010027 (PMC5876582; doi:10.3390/vetsci5010027)
Supplement: Supplementary file 1 [file vetsci-05-00027-s001.zip › vetsci-273655 Supplementary For Final/Supplemental File Figure S1.docx]

| 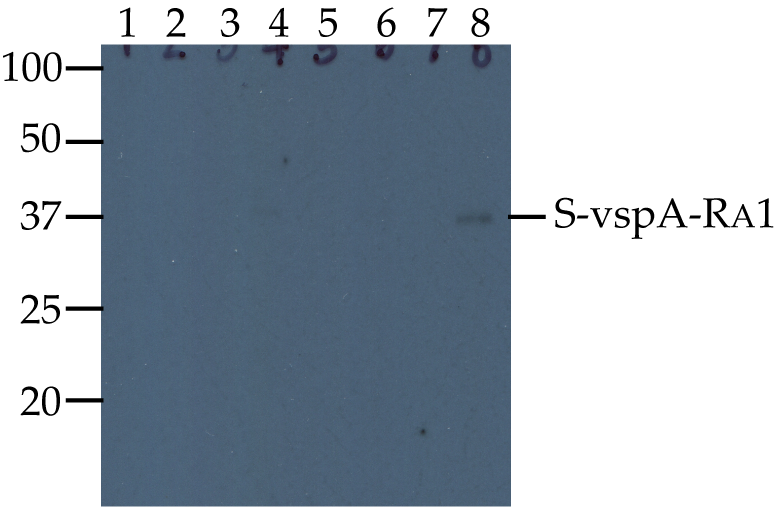 | 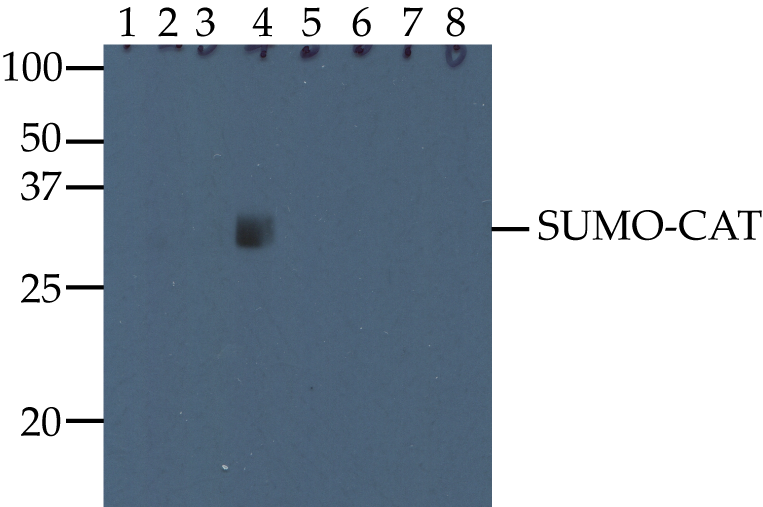 |
| --- | --- |
| (**A**) | (**B**) |
| 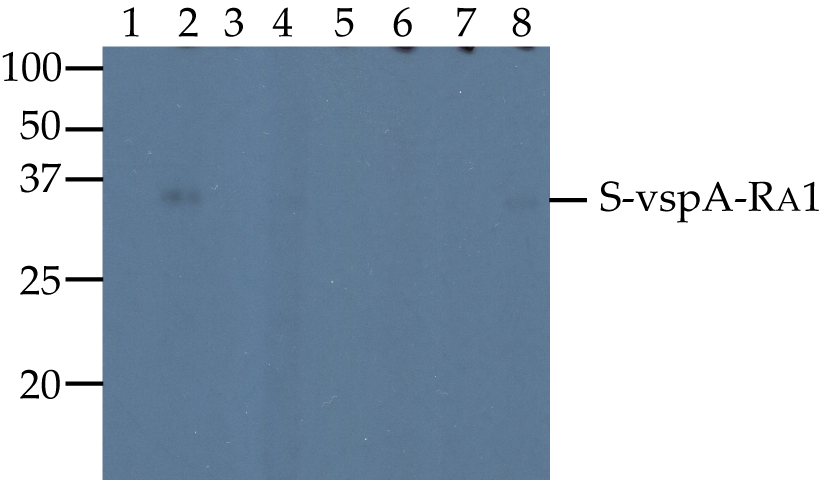 | 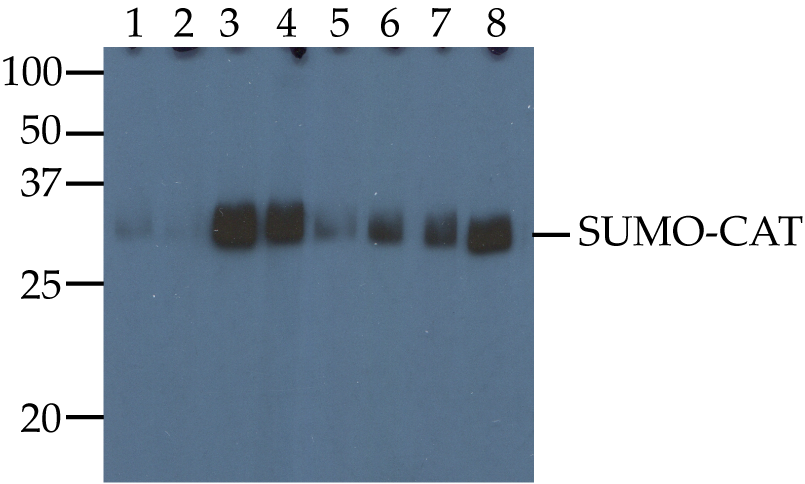 |
| (**C**) | (**D**) |
| 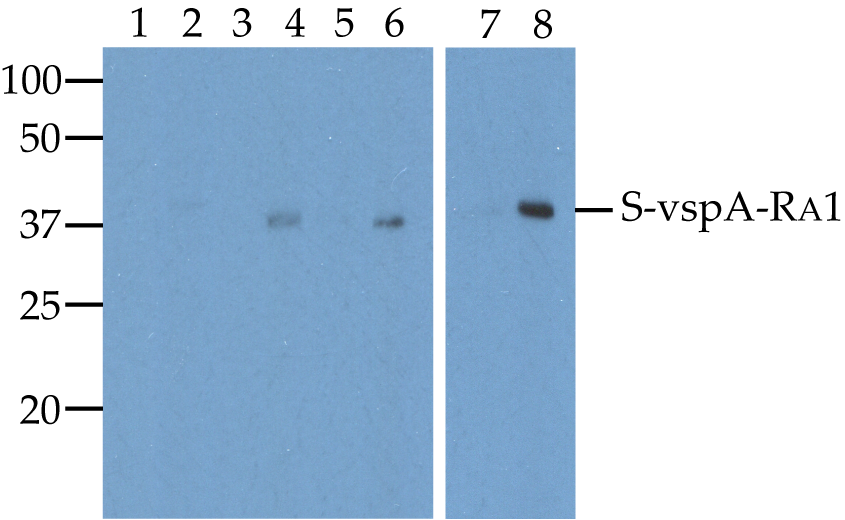 | 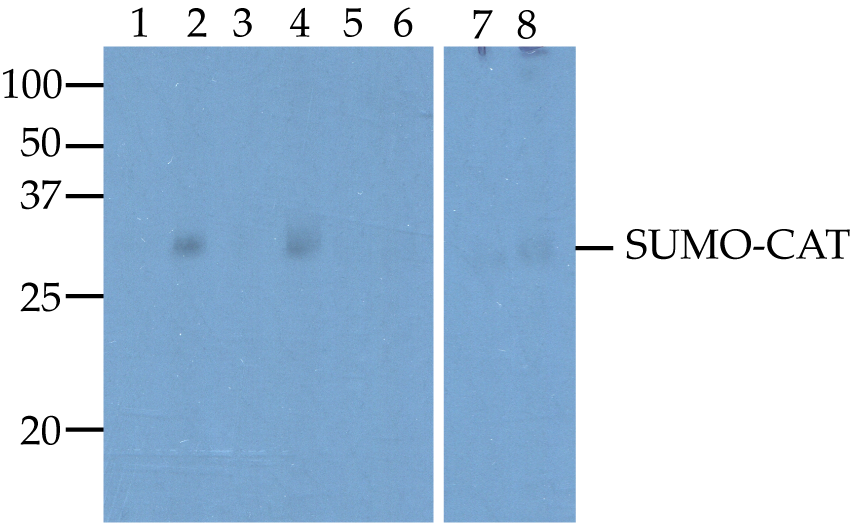 |
| (**E**) | (**F**) |
| 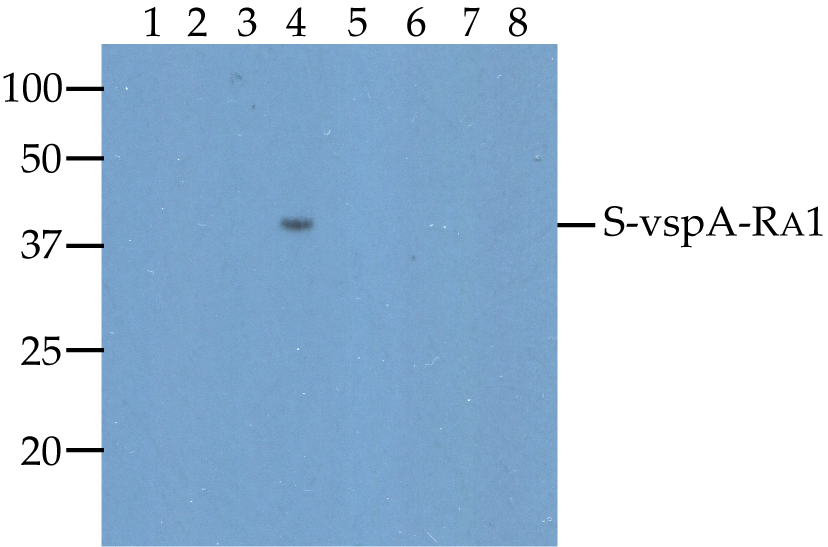 | 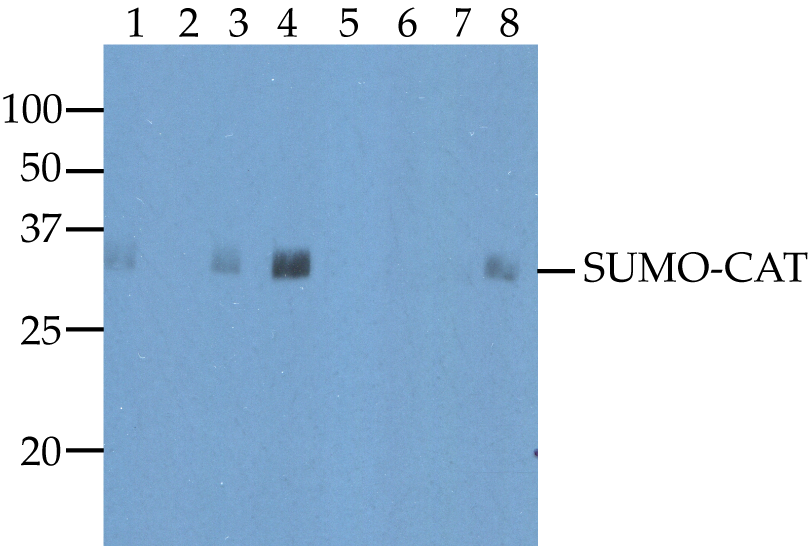 |
| (**G**) | (**H**) |
| 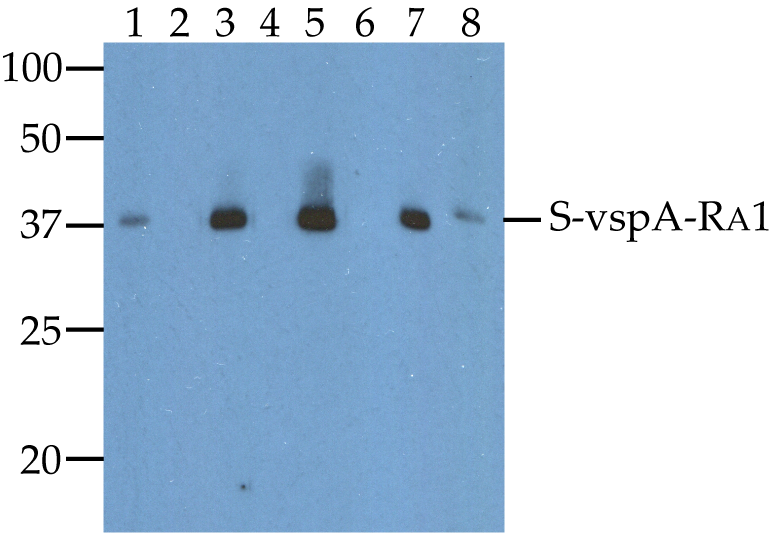 | 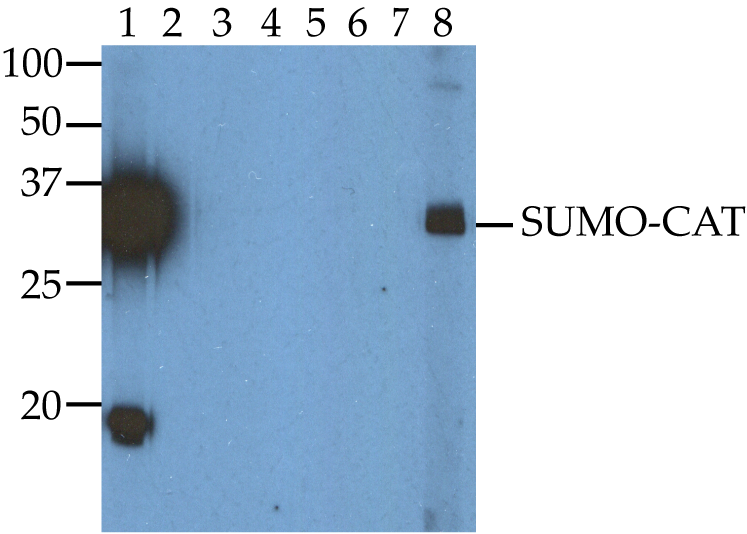 |
| (**I**) | (**J**) |

**Figure S1.** Western blot analyses of selected Australian paired sera (n = 16) and ELISA control samples using the S-vspA-RA1 and SUMO-CAT polypeptides. Animal numbers are shown with postscripts indicated the day of serum collection, Day 0 (D0) and Day 24 (D24). The 100 kDa, 50 kDa, 37 kDa, 25 kDa and 20 kDa molecular markers (kDa) are shown. (**A**) Reactivity to S-vspA-R_A_1 and (**B**) Reactivity to SUMO-CAT: Lane 1 APCAH3_D0; Lane 2 APCAH3_D24; Lane 3 APCAH5_D0; Lane 4 APCAH5_D24; Lane 5 APCAH7_D0; Lane 6 APCAH7_D24; Lane 7 APCAH8_D0; Lane 8 APCAH8_D24. (**C**) Reactivity to S-vspA-R_A_1 and (**D**) Reactivity to SUMO-CAT: Lane 1 APCAH9_D0; Lane 2 APCAH9_D24; Lane 3 APCAH10_D0; Lane 4 APCAH10_D24; Lane 5 APCAH13_D0; Lane 6 APCAH13_D24; Lane 7 APCAH18_D0; Lane 8 APCAH18_D24. (**E**) Reactivity to S-vspA-R_A_1 and
(**F**) Reactivity to SUMO-CAT: Lane 1 APCAH20_D0; Lane 2 APCAH20_D24; Lane 3 APCAH21_D0; Lane 4 APCAH21_D24; Lane 5 APCAH22_D0; Lane 6 APCAH22_D24; Lane 7 APCAH23_D0; Lane 8 APCAH23_D24. (**G**) Reactivity to S-vspA-R_A_1 and (**H**) Reactivity to SUMO-CAT: Lane 1 APCA24_D0; Lane 2 APCAH24_D24; Lane 3 APCAH26_D0; Lane 4 APCAH26_D24; Lane 5 APCAH30_D0; Lane 6 APCAH30_D24; Lane 7 APCAH31_D0; Lane 8 APCAH31_D24. (**I**) Reactivity to S-vspA-R_A_1 and
(**J**) Reactivity to SUMO-CAT of selected control antibodies: Lane 1 Hexa-histidine monoclonal antibody; Lane 2 SMYC09128 Negative; Lane 3 SMYC09128 Positive; Lane 4 MYC13K26 Negative; Lane 5 MYC13K26 Positive; Lane 6 SMYC13K26 Negative; Lane 7 SMYC13K26 Positive; Lane 8 IBRPM12F04 Positive.

© 2018 by the authors. Submitted for possible open access publication under the
terms and conditions of the Creative Commons Attribution (CC BY) license (http://creativecommons.org/licenses/by/4.0/).
