# Supplementary material for: The Performance of Three Immune Assays to Assess the Serological Status of Cattle Experimentally Exposed to Mycoplasma bovis
Source: Vet Sci. 2018 Mar 8;5(1):27. doi: 10.3390/vetsci5010027 (PMC5876582; doi:10.3390/vetsci5010027)
Supplement: Supplementary file 1 [file vetsci-05-00027-s001.zip › vetsci-273655 Supplementary For Final/Supplemental File Table S1.docx]

**Table S1.** Monoclonal antibodies used in this study and the *Mycoplasma bovis* variable surface proteins (Vsp) recognised. The reported molecular sizes of each Vsp are shown in parenthesis.

| Monoclonal Antibody | Specificity | Reference |
| --- | --- | --- |
| 1E5 | VspA (63–67 kDa),  VspB (35–46 kDa), and VspC (75 kDa) | Behrens et al. [6] and Lysnyansky et al. [29] |
| 2A8 | VspC  VspO (40 kDa) | Beier et al. [7] |
| 9F1 | VspF (55 kDa) | Lysnyansky et al. [30] |
| 4D7 | VspA, VspB and VspC ^1^ | Beier et al. [7] |
| 6E5 | VspA | Beier et al. [7] |

^1^ Distinct epitope from 1E5

© 2018 by the authors. Submitted for possible open access publication under the
terms and conditions of the Creative Commons Attribution (CC BY) license (http://creativecommons.org/licenses/by/4.0/).
