# Supplementary material for: The Performance of Three Immune Assays to Assess the Serological Status of Cattle Experimentally Exposed to Mycoplasma bovis
Source: Vet Sci. 2018 Mar 8;5(1):27. doi: 10.3390/vetsci5010027 (PMC5876582; doi:10.3390/vetsci5010027)
Supplement: Supplementary file 1 [file vetsci-05-00027-s001.zip › vetsci-273655 Supplementary For Final/Supplemental File Table S1.pdf]

veterinary sciences

Table S1. Monoclonal antibodies used in this study and the *Mycoplasma bovis* variable surface proteins (Vsp) recognised. The reported molecular sizes of each Vsp are shown in parenthesis.

Monoclonal Antibody	Specificity	Reference
1E5	VspA (63–67 kDa), VspB (35–46 kDa), and VspC (75 kDa)	Behrens et al. [6] and Lysnyansky et al. [29]
2A8	VspC VspO (40 kDa)	Beier et al. [7]
9F1	VspF (55 kDa)	Lysnyansky et al. [30]
4D7	VspA, VspB and VspC [1]	Beier et al. [7]
6E5	VspA	Beier et al. [7]

[1] Distinct epitope from 1E5

© 2018 by the authors. Submitted for possible open access publication under the terms and conditions of the Creative Commons Attribution (CC BY) license (http://creativecommons.org/licenses/by/4.0/).
